# Combined Effects of Micro- and Nanoplastics at the Predicted Environmental Concentration on Functional State of Intestinal Barrier in *Caenorhabditis elegans*

**DOI:** 10.3390/toxics11080653

**Published:** 2023-07-28

**Authors:** Yu Wu, Xiaochao Tan, Xian Shi, Peiyu Han, Huanliang Liu

**Affiliations:** Environment and Health Research Division, Public Health Research Center, Wuxi School of Medicine, Jiangnan University, Wuxi 214122, China

**Keywords:** combined toxicity, nanoplastics, microplastics, environmentally relevant concentrations, intestinal barrier, *Caenorhabditis elegans*

## Abstract

The possible toxicity caused by nanoplastics or microplastics on organisms has been extensively studied. However, the unavoidably combined effects of nanoplastics and microplastics on organisms, particularly intestinal toxicity, are rarely clear. Here, we employed *Caenorhabditis elegans* to investigate the combined effects of PS-50 (50 nm nanopolystyrene) and PS-500 (500 nm micropolystyrene) at environmentally relevant concentrations on the functional state of the intestinal barrier. Environmentally, after long-term treatment (4.5 days), coexposure to PS-50 (10 and 15 μg/L) and PS-500 (1 μg/L) resulted in more severe formation of toxicity in decreasing locomotion behavior, in inhibiting brood size, in inducing intestinal ROS production, and in inducting intestinal autofluorescence production, compared with single-exposure to PS-50 (10 and 15 μg/L) or PS-500 (1 μg/L). Additionally, coexposure to PS-50 (15 μg/L) and PS-500 (1 μg/L) remarkably caused an enhancement in intestinal permeability, but no detectable abnormality of intestinal morphology was observed in wild-type nematodes. Lastly, the downregulation of *acs-22* or *erm-1* expression and the upregulation expressions of genes required for controlling oxidative stress (*sod-2*, *sod-3*, *isp-1*, *clk-1*, *gas-1*, and *ctl-3*) served as a molecular basis to strongly explain the formation of intestinal toxicity caused by coexposure to PS-50 (15 μg/L) and PS-500 (1 μg/L). Our results suggested that combined exposure to microplastics and nanoplastics at the predicted environmental concentration causes intestinal toxicity by affecting the functional state of the intestinal barrier in organisms.

## 1. Introduction

Due to the insufficient recycling and reusing system, large quantities of plastics have been randomly released into ecosystems, including the terrestrial ecosystem, marine ecosystem, and freshwater ecosystem [1]. Generally, plastics refer to various morphological debris that originates from multiple plastic product degradation [2]. Such plastic debris can be further degraded into microplastic particles (<5 mm) or nanoplastic particles (<100 nm) [3,4]. As is well known, particle size plays a dominant role in determining particle biodistribution and biotoxicity [5]. Long-term exposure to small sizes of nanopolystyrene particles (20 nm) could cause more severe exposure risk in causing transgenerational toxicity than larger sizes of nanopolystyrene particles (100 nm) in nematodes [6]. The particle size is inversely proportional to micro- and nanoplastics absorption in organisms [7,8,9]. In vertebrates, polystyrene microplastic exposure induced testis developmental disorder and affected male fertility in mice [10]. In addition, alteration in functional group modification of nanopolystyrene particles could also cause different toxicity formations. For example, sulfonate- or amino-modified nanopolystyrene could cause more serve neurotoxicity or reproductive toxicity in *Caenorhabditis elegans* [11,12]. UV-aged microplastics induced neurotoxicity via regulating the neurotransmission in larval zebrafishes [13]. However, the detected toxicity in organisms was largely based on single-exposure to micro- or nanoplastics. In a real environment, the combined toxicity caused by coexposure to microplastics and nanoplastics in multidimensional sizes is unavoidably imposed on organisms [14,15]. Currently, most studies have focused on coexposure to microplastics and persistent organic pollutants or heavy metal. For example, the combined exposure to MPs and Cd may exert synergistic toxic effects on crucian carp [16]. Coexposure to nanopolystyrene particles and BPA exhibited more serve toxicity on histopathology, oxidative stress, immune function, and intestinal microbiota in channel catfish [17]. Reportedly, the interplay among different-sized plastics may enhance the cellular uptake efficiency [18]. That is, the unexpected interplay may induce more severe biodistribution and biotoxicity [19]. Currently, Huang et al. showed that coexposure to micro- and nanoplastics at 2.5–50 mg/kg body weight resulted in more severe intestinal barrier dysfunction in mice, compared to single-exposure to micro- or nanoplastics [19]. Nevertheless, the research on the combinational effects of plastics particles with multidimensional size at the estimated environmental concentrations on organisms are largely limited. Moreover, the estimated environmental nanoplastics concentrations were speculated to be ≤15 μg/L for 50 nm nanoscale and ≤1 μg/L for 500 nm [20,21].

*Caenorhabditis elegans* is a well-established, wonderful animal model owing to its typical properties [22]. Additionally, *C. elegans* exhibits enhanced sensitivity to various environmental toxicants or stresses, which makes it a successful model in toxicological evaluation and signaling studies. Some beneficial sublethal endpoints, (e.g., locomotion behavior, brood size, oxidative stress, and intestine permeability) were evaluated as indicators of the potential toxicity of multiple toxicants [6,23,24]. In nematodes, prolonged single-exposure to micro- or nanoplastics particles can both cause neurotoxicity and reproductive toxicity [11,25,26]. Additionally, long-term contact (from L1-larvae to adult day-1) with nanopolystyrene particles (≥10 μg/L) can induce intestinal reactive oxygen species (ROS) production, reduced locomotor behavior, and decreased brood size [27]. The gut, as an important primary target organ, is critical in responding to environmental toxicants, particularly plastic particles [28,29,30]. Nevertheless, the possible combined effects of micro- and nanoplastics at the estimated environmental concentration on nematodes, especially intestinal toxicity, remain unclear.

Herein, our aim was to examine the combined effect of nanopolystyrenes and micropolystyrenes at the predicted environmental concentration on the functional state of the intestinal barrier in *Caenorhabditis elegans*. In this study, the 50 nm nanopolystyrene (PS-50) and 500 nm micropolystyrene (PS-500) were chosen as the test materials. We measured the locomotion behavior, brood size, intestinal reactive oxygen species (ROS) production, intestinal autofluorescence, and used scanning electron microscopy (SEM) to assess PS-50 and PS-500. Finally, we hypothesized that coexposure to micro- and nanoplastics at estimated environmentally significant concentrations could potentially induced a more severe deterioration in the functional state of the intestinal barrier than single-exposure to micro- or nanoplastics. The associated cellular or molecular basis for this combined effect was also presented.

## 2. Materials and Methods

### 2.1. Nanopolystyrene Characterizations

**Figure 1:**
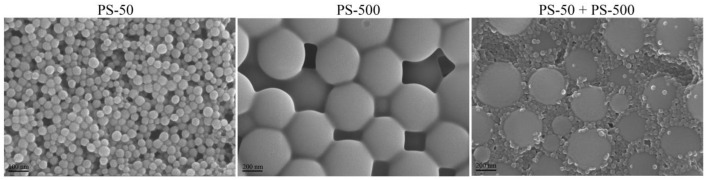
Physicochemical properties of PS-50 and PS-500. TEM images of PS-50, PS-500, and mixture of PS-50 and PS-500 in K-medium before the sonication.

The 50 nm nanopolystyrene (PS-50) and 500 nm micropolystyrene (PS-500) were obtained from Janus New-Materials Co., Ltd., Nanjing, China. Dynamic light scattering (DLS) analysis further indicated that the sizes of the examined PS-50 and PS-500 were 49.46 ± 2.3 nm and 502.55 ± 3.1 nm, respectively. The images from the scanning electron microscope (SEM) showed the spherical morphology of PS-50 and PS-500 (Figure 1). The zeta potentials of PS-50 were −9.447 ± 0.224 mV and PS-500 was −9.172 ± 0.426. The working suspensions were made by diluting the stock solution with liquid K-medium. Before exposure, a 30 min sonication at 40 kHz (100 W) was performed to make the working suspensions monodisperse. The FTIR spectrum of PS-50 and PS-500 is shown in Supplementary Figure S1.

### 2.2. Animal Maintenance 

*C. elegans* (wild type, Bristol strain N2) were acquired from School of Medicine, Southeast University (Nanjing, China, accessed on 16 May 2022) and cultured on nematode growth medium plates containing *Escherichia coli* OP50, the food source, at 20 °C without light [31]. To extract worms at the same developmental stage, we prepared a bleaching solution (2% HOCl, 0.45 M NaOH) to synchronize pregnant worms over a 5 min period to discharge enough eggs [32]. These worms were then rinsed thrice with K-medium (0.032 M KCl, 0.051 M NaCl) [33], before transfer to fresh NGM plates with OP50 feeding, and incubated at 20 °C for 24 h without light to acquire L1 stage worms. 

### 2.3. Exposure and Assessment Endpoints 

The estimated environmental PS-50 concentrations were chosen as 5, 10, and 15 μg/L [20,21]. The selected working concentrations of PS-500 were 0.1 and 1 μg/L [20]. Long-term treatment (L1-larvae to adult day-1) was used to treat the examined worms and the exposure suspensions were introduced to *E. coli OP50* (~4 × 106 colony-forming units (CFUs)). During the exposure period, the suspensions were replenished daily. Herein, some endpoints were performed to detect the potential combined toxicity between PS-50 and PS-500 at the predicted environmental concentration on nematodes.

Locomotor behavior, including head thrashing and body bending, was employed to assess the motor neuronal operative status as described [6]. Head thrashes were quantified according to the posterior bulb direction (y-axis) alterations, with an assumption that the x-axis was the traveling direction [24,32]. Body bends were quantified as the midbody bending directional alterations [24,32]. For individual contacts, 40 worms were analyzed.

Brood size was used to assess the reproductive capacity [34]. The total number of the progeny during the development beyond the eggs was counted [35]. For each exposure treatment, 20 worms were analyzed. 

Intestinal ROS synthesis was assessed as reported earlier [31]. *C. elegans* were exposed to 1 μM 5′,6′-chloromethyl-2′,7′-dichlorodihydro-fluorescein diacetate (CM-H2DCFDA), prior to a 3 h incubation without light. The tested organisms were rinsed thrice in K-medium (0.032 M KCl, 0.051 M NaCl), and then mounted on a 2% agar pad to evaluate intestinal fluorescent ROS production, using a fluorescence microscope with an excitation wavelength at 488 nm and an emission filter of 510 nm. The relative fluorescence intensity representing intestinal ROS production was semiquantified in relation to the intestinal autofluorescence. Overall, 40 animals were assessed per group, and each group was tested three times.

Intestinal autofluorescence brought on by the lipofuscin-mediated lysosomal deposition refers to the aging process in worms [35]. After contact, we mounted nematodes on a 2% agar plates and measured them under the DAPI channel of a microscope. The fluorescence intensity was quantified using Image J.

Intestinal permeability reflecting the functional state of the intestine barrier was routinely performed to exhibit the intestine damage induced by environmental pollutants [29]. Erioglaucine disodium (5.0%) was diluted using K-medium with OP50 addition as a food source [23]. Then, the exposed worms were soaked in the prepared staining liquid for 3 h in the dark. After staining, we used K-medium to wash the nematodes six times. Image capture was performed under the bright field. Overall, 30 animals were employed for individual treatments, with three biological replicates per treatment. 

### 2.4. Quantitative Real-Time Polymerase Chain Reaction (qRT-PCR)

Total RNAs were prepared with the help of Trizol (Sigma-Aldrich, Milwaukee, Germany). Then, cDNA was converted (37 °C, 15 min; 42 °C, 60 min; and 70 °C, 15 min) in a Mastercycler gradient PCR system. The relative gene expressions (*act-5*, *pkc-3*, *acs-22*, *erm-1*, *hmp-2*, *sod-1*, *sod-2*, *sod-3*, *sod-4*, *sod-5*, *mev-1*, *isp-1*, *clk-1*, *gas-1*, *ctl-1*, *ctl-2*, and *ctl-3*) were recorded using the StepOnePlus™ real-time PCR system. All gene expressions were normalized to *tba-1* (a reference gene). All experiments were conducted three times. The employed primer sequences are provided in Supplementary Table S1.

### 2.5. Statistical Analyses

SPSS 12.0 was employed for all data analyses, and data was provided as mean ± standard derivation (SD). Intergroups assessment utilized the one-way or two-way ANOVA with multiple-factor comparison followed by a post hoc test. A probability level of 0.01 (**) was set as the significance threshold.

## 3. Results

### 3.1. Combined Effects of PS-50 and PS-500 on Locomotion Behavior and Brood Size

**Figure 2:**
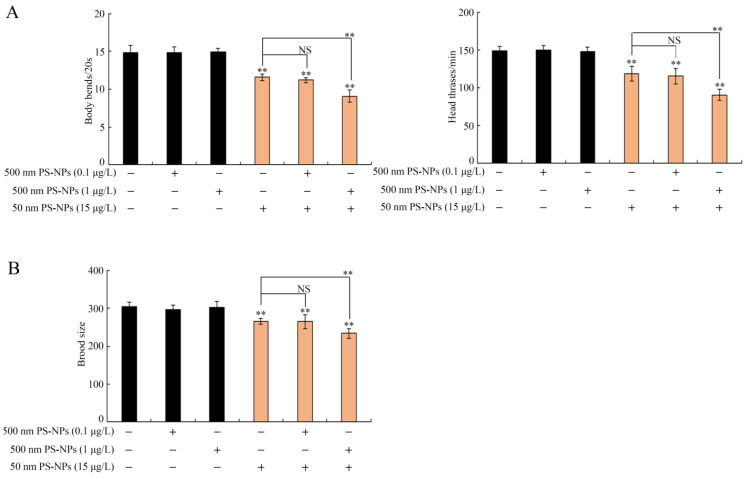
Combined effects between PS-50 and PS-500 on brood size (**A**) and locomotor behavior (**B**) in nematodes. Long-term exposure was provided from L1-larvae to adult day-1. “+”, with; “−”, lacking. Control, without polystyrene particle treatment. Bars denote means ± SD. ** *p* < 0.01 vs. control (if not specially indicated); NS, no significant difference.

PS-500 (0.1 and 1 μg/L) exposure showed no difference in brood size or locomotion behavior (Figure 2). Similarly, PS-50 (5 μg/L) did not obviously alter brood size or locomotion behavior (Supplementary Figure S2). However, PS-50 (10 and 15 μg/L) notably decreased brood size and locomotion behavior (Figure 2 and Figure S2). Coexposure to PS-500 (0.1 μg/L) and PS-500 (10 and 15 μg/L) did not cause any adverse effect on locomotion behavior and brood size in worms (Figure 2 and Figure S2). However, PS-500 (1 μg/L) notably enhanced the PS-50 (10 and 15 μg/L) toxicity in decreasing brood size and inhibiting locomotion behavior in a dose-dependent manner (Figure 2 and Figure S2). Interestingly, combined exposure of PS-500 (1 μg/L) and PS-50 (5 μg/L) noticeably reduced locomotion behavior rather than brood size in wild-type nematodes (Supplementary Figure S2).

### 3.2. Combined Effects of PS-50 and PS-500 on Intestinal Morphology

**Figure 3:**
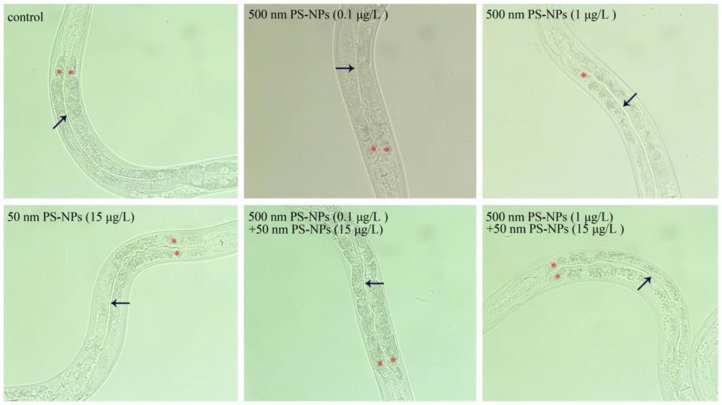
Combined effects between PS-50 and PS-500 exposures on intestinal morphology. Arrowheads indicate the intestinal lumen. Asterisks indicate the intestinal cells. Long-term exposure was provided from L1-larvae to adult day-1.

After prolonged exposure, we first examined intestinal morphology to detect the potential toxicity on intestinal structure. Whether coexposure or single-exposure to PS-50 and PS-500, it did not all cause remarkable changes in the intestinal lumen (Figure 3). Furthermore, no aberration was confirmed for the intestinal cells in nematodes co- or single-exposed to PS-50 and PS-500 (Figure 3). Therefore, the interplay of PS-50 and PS-500 at the predicted environmental concentration did not influence the intestinal morphology in worms.

### 3.3. Combined Effects of PS-50 and PS-500 on Intestinal Permeability

**Figure 4:**
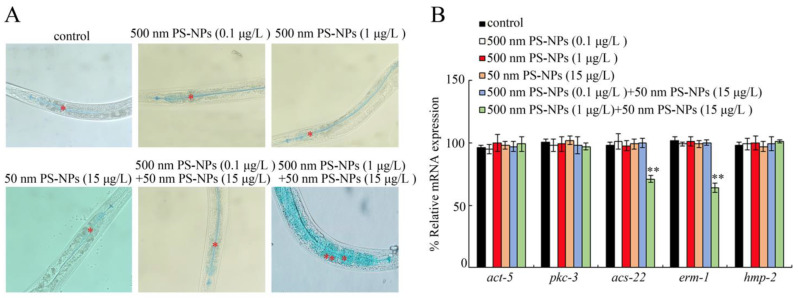
Combined effects between PS-50 and PS-500 exposure on intestinal permeability (**A**) and intestinal-barrier-related gene expression (**B**). The intestinal cells (**) and intestinal lumen (*) are marked by asterisks. qRT-PCR analysis was conducted with RNA isolated from 30 intact intestines. ** *p* < 0.01 vs. control.

According to our erioglaucine disodium staining method, under normal conditions, the blue dye only stained the intestinal lumen (Figure 4A and Figure S3). Single-exposure to PS-50 (5, 10, and 15 μg/L) or PS-500 (0.1 and 1 μg/L) did not produce any alteration in intestinal permeability (Figure 4A and Figure S3). Moreover, coexposure to PS-50 (5, 10, and 15 μg/L) and PS-500 (0.1 μg/L), or to PS-50 (5 and 10 μg/L) and PS-500 (1 μg/L), did not induce any abnormality in intestinal permeability (Figure 4A and Figure S3). However, combined treatment with PS-50 (15 μg/L) and PS-500 (1 μg/L) strongly enhanced intestinal permeability, which was evidenced by the translocation and accumulation of the blue dye into intestinal cells through the intestinal barrier (Figure 4A). 

We further detected the expressions of the genes involved in regulating the functional state of the intestinal barrier. Single-expoure to PS-50 (15 μg/L) or PS-500 (0.1 and 1 μg/L) did not influence intestinal *act-5*, *pkc-3*, *acs-22*, *erm-1*, and *hmp-2* contents (Figure 4B). Similarly, coexposure to PS-50 (15 μg/L) and PS-500 (0.1 μg/L) also did not modulate intestinal *act-5*, *pkc-3*, *acs-22*, *erm-1*, and *hmp-2* contents (Figure 4B). However, coexosure to PS-50 (15 μg/L) and PS-500 (1 μg/L) strongly suppressed intestinal *acs-22* and *erm-1* expressions (Figure 4B). 

### 3.4. Combined Effects of PS-50 and PS-500 on Intestinal Autofluorescence

**Figure 5:**
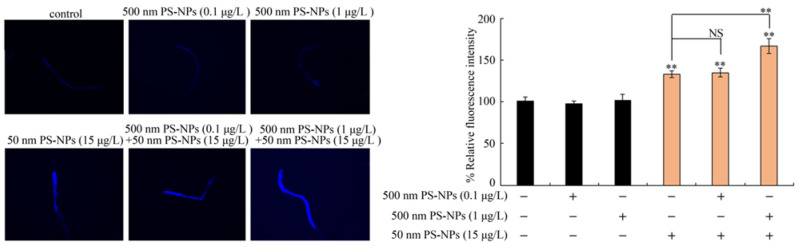
Combined effects between PS-50 and PS-500 exposures on intestinal autofluorescence. Long-term exposure was provided from L1-larvae to adult day-1. “+”, with; “−”, lacking. Control, without polystyrene particle treatment. Bars denote means ± SD. ** *p* < 0.01 vs. control (if not specially indicated); NS, no significant difference.

Using intestinal autofluorescence as an endpoint, PS-500 (0.1, 1 μg/L) did not significantly affect intestinal autofluorescence production (Figure 5 and Figure S4). Similarly, PS-50 (5 μg/L) did not significantly induce enhancement of intestinal autofluorescence either (Supplementary Figure S4). However, PS-50 (10, 15 μg/L) exposure produced considerable intestinal autofluorescence (Figure 5 and Figure S4). In the coexposure groups, PS-500 (0.1 μg/L) did not remarkably influence the toxicity of PS-50 (10 and 15 μg/L) in generating intestinal autofluorescence (Figure 5 and Figure S4). Nevertheless, PS-500 (1 μg/L) notably enhanced PS-50 (10 and 15 μg/L) toxicity in inducing intestinal autofluorescence synthesis in a dose-dependent manner (Figure 5 and Figure S4). Noteworthily, combined interplay between PS-500 (1 μg/L) and PS-50 (5 μg/L) failed to produce observable improvement in intestinal autofluorescence in wild-type nematodes (Supplementary Figure S4).

### 3.5. Combined Effects of PS-50 and PS-500 on Induction of Oxidative Stress

**Figure 6:**
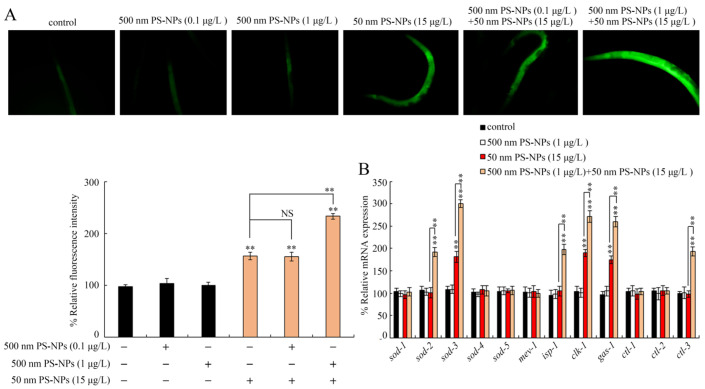
Combined effects between PS-50 and PS-500 exposures on intestinal oxidative stress (**A**) and oxidative-stress-related gene expression (**B**). Long-term exposure was provided from L1-larvae to adult day-1. “+”, with; “−”, lacking. Control, without polystyrene particle treatment. Bars denote means ± SD. ** *p* < 0.01 vs. control (if not specially indicated); NS, no significant difference.

The reactive oxygen species (ROS) is a byproduct of the mitochondria-based electron transport chain reaction, and it positively modulates cellular senescence and organ dysfunction via inducting oxidative damages to DNA, proteins, and lipids [36]. PS-500 (0.1 and 1 μg/L) did not produce excessive intestinal ROS to the control (Figure 6A and Figure S5). Meanwhile, PS-50 (5 μg/L) did not strongly enhance intestinal ROS synthesis (S6). Nevertheless, PS-50 (10 and 15 μg/L) strongly enhanced intestinal ROS synthesis (Figure 6A and Figure S5). In coexposure groups, PS-500 (0.1 μg/L) did not remarkably influence PS-500 (10 and 15 μg/L) toxicity, which modulated intestinal ROS synthesis (Figure 6A and Figure S5). However, PS-500 (1 μg/L) notably increased PS-50 (10 and 15 μg/L) toxicity, as evidenced by the dose-dependent enhancement of intestinal ROS synthesis in a dose-dependent manner (Figure 6A and Figure S5). Importantly, combined exposure to PS-500 (1 μg/L) and PS-50 (5 μg/L) also produced considerable intestinal ROS in wild-type nematodes (Supplementary Figure S5).

### 3.6. Combined Effects of PS-50 and PS-500 on Molecular Basis of Oxidative Stress

Superoxide dismutase (SOD-1–5) and catalase (CTL1–3) are critical contributors to the antioxidation-based defense response in nematodes, and mitochondrial complexes (ISP-1, CLK-1, MEV-1, and GAS-1) participate in oxidative stress activation [37,38,39]. Among these examined genes, PS-500 (1 μg/L) did not alter the content of any analyzed genes. However, PS-500 (15 μg/L) remarkably enhanced *sod-3*, *clk-1*, and *gas-1* expressions (Figure 6B). Furthermore, coexposure to PS-50 (1 μg/L) and PS-500 (15 μg/L) further enhanced the *sod-2*, *sod-3*, *isp-1*, *clk-1*, *gas-1*, and *ctl-3* levels (Figure 6B). 

## 4. Discussion

To date, sufficient evidences provided by previous studies have raised that prolonged exposure to micro- or nanoplastics results in severe multiorgan toxicity in environmental organisms, including neurotoxicity, reproductive toxicity, and immunotoxicity in environmental organisms [11,26,40,41,42]. However, the combined effects of microplastics and nanoplastics, especially at predicted environmental relevant concentration, on organisms remains unclear. Herein, locomotion behavior and brood size, as sensitive ecological indicators, could more effectively reflect the potential biotoxicity of environmental pollutants [43,44]. Firstly, we used sensitive endpoints (brood size and locomotion behavior) to examine the combinational effects between PS-50 and PS-500 in nematodes. We revealed that long-term PS-500 (1 μg/L) exposure could prominently enhance PS-50 (15 μg/L) toxicity in diminishing locomotion behavior and brood size (Figure 2). That is, although PS-500 (1 μg/L) alone would not cause toxicity, a synergistic effect between PS-500 (1 μg/L) and PS-50 (15 μg/L) on nematodes can be formed. That is, coexposure to micro- and nanoplastics at estimated environmentally significant concentrations showed more serve toxicity on organisms in the environment. As reported, prolonged exposure to nanopolystyrene particles (100 nm) at estimated environmentally significant concentrations (1 μg/L) did not promote toxicity in nematodes [27]. The above observation suggests that the interplay of PS-50 and PS-500 might cause more severe toxicity on motor neuronal activity in wild-type nematodes compared to that in separate exposure groups. Besides this, no detectable alteration in brood size was detected in worms cotreated with PS-500 (1 μg/L) and PS-50 (5 μg/L) (S2B), which implies that brood size may not be so sensitive in examining the improvement in combined toxicity. Differently, brood size showed a more susceptible property than locomotion behavior in controlling MC-LR toxicity [45], likely because of differences in sensitivity to different toxicants or exposure durations.

The broadened intestinal lumen is frequently used as an indicator to reflect the abnormality in intestinal morphology [23,35]. Recently, some environmental stress, such as simulated microgravity, can alter the intestinal morphology [23]. In contrast, neither single-exposure or coexposure to PS-500 (0.1, 1 μg/L) and PS-50 (15 μg/L) resulted in the noticeable structural changes in the intestinal lumen or intestinal cells in nematodes (Figure 3). That is, the interplay of PS-50 and PS-500 at environmental relevant concentration was not sufficient to damage the intestinal morphology. Similarly, exposure to graphene oxide or 6-PPDQ in the range of μg/L did not also induce the abnormality of intestinal morphology [35,46]. 

Intestinal permeability is usually performed to assess the functional state of the intestinal barrier [35,47]. Different from the observation of intestinal morphology, we observed remarkable improvement in intestinal permeability only after coexposure in the PS-50 (15 μg/L) and PS-500 (1 μg/L) group rather than that in other groups (Figure 4A and Figure S3). Subacute PS-MPs exposure also caused a hyperpermeable state of the intestinal barrier, indicating that PS-MPs exposure resulted in intestinal damage to nematodes [30]. Interestingly, the blue dye was translocated from the intestinal lumen to intestinal cells, but the blue dye was not further translocated into the body cavity in nematodes coexposed to PS-50 (15 μg/L) and PS-500 (1 μg/L) (Figure 4A). The results imply that combined exposure to PS-50 and PS-500 at environmental relevant concentration generated severe intestine toxicity in enhancing intestinal permeability and damaging the functional state of the intestinal barrier rather than single-exposure to PS-50 or PS-500. More importantly, Liang et al. also showed that polystyrene micro- and nanoplastics synergistically induced intestinal barrier dysfunction via an ROS-mediated epithelial cell apoptotic pathway in mice [19]. Additionally, nanopolystyrene at the estimated environmental concentration can enhance microcystin-LR toxicity via intestinal destruction in *Caenorhabditis elegans* [48]. 

Some crucial regulators are involved in controlling for intestinal permeability. Similar to the observation of intestinal permeability, coexposure to PS-50 (15 μg/L) and PS-500 (1 μg/L) remarkably decreased the intestinal *acs-22* and *erm-1* expressions (Figure 4B), which may be intricately linked to the detectable rise in intestinal permeability. In *C. elegans*, ACS-22 encodes a protein homologous to mammalian fatty acid transport protein and is necessary for controlling intestinal permeability [23]. ERM-1 encodes an Ezrin–radixin–moesin protein needed for maintaining the intestinal barrier functional state [23]. Hence, the underlying mechanism of intestinal barrier function is potentially changed by coexposure to PS-50 and PS-500 at the predicted environmental concentration. Furthermore, no significant changes in intestinal *act-5*, *pkc-3*, and *hmp-2* contents were confirmed (Figure 4B), which were also important contributors to maintain the normally functional state of intestinal barrier in nematodes.

To explore the underlying intracellular mechanism required for the PS-50 and PS-500 coexposure-mediated modulation of intestine toxicity, we proposed oxidative stress and intestinal autofluorescence in this study. Intestinal autofluorescence is generated via lysosomal deposition of lipofuscin, which accumulates over time in aging nematodes [49]. Oxidative stress is the major contributor to the toxicity formation of toxicants [50]. Prolonged exposure to PS-500 (1 μg/L) enhanced PS-50 (10 and 15 μg/L) toxicity in inducing intestinal autofluorescence and intestinal ROS synthesis (Figure 5 and Figure 6A, Figures S4 and S5); this observation further supports the more severe intestine toxicity caused by the combined exposure to PS-50 and PS-500 at environmentally relevant concentration. Similarly, exposure to polystyrene microplastics (1 μm) at 72 h also induced oxidative stress and intestinal injury in nematode *Caenorhabditis elegans* [30], which implies the close association between oxidative stress and intestine toxicity caused by polystyrene microplastic exposure. Intriguingly, coexposure to PS-500 (1 μg/L) and PS-50 (5 μg/L) produced a noticeable rise in intestinal ROS synthesis rather than intestinal autofluorescence synthesis (Supplementary Figures S4 and S5). The reason may be that intestinal ROS synthesis is more sensitive than intestinal autofluorescence. Furthermore, nanopolystyrene particles at the estimated environmentally significant concentration enhanced the environmental ENMs (TiO_2_-NPs) toxicity on nematodes by diminishing locomotion behavior and enhancing intestinal ROS synthesis [47].

In *C.elegans*, SODs, catalases, and mitochondrial complex components act as crucial regulators to maintain the oxidative stress balance [51,52]. Compared to the control group, PS-500 (1 μg/L) did not produce any alteration among the examined genes required for controlling ROS production (Figure 6B). Differently, obvious rises in *sod-3, clk-1,* and *gas-1* expressions occurred in worms exposed to PS-50 (15 μg/L). More interestingly, coexposure to PS-500 (1 μg/L) and PS-50 (15 μg/L) further dramatically enhanced the *sod-2, sod-3, isp-1, clk-1, gas-1,* and *clt-3* expressions. SOD-2 and SOD-3 cooperate with CTL-3 to modulate the oxidation–antioxidation system in nematodes [23,35]. ISP-1, CLK-1, and GAS-1 localize to the mitochondria electron transport chain to sever as important components to control intestinal ROS production [47]. Environmentally, these observations provided the indirect evidence for the more severe toxicity formation in nematodes coexposed to PS-50 and PS-500 at environmentally relevant concentration compared with single-exposure to PS-50 or PS-500. It is implied that nanoplastic collaboration with microplastics synergistically contributes to the remarkable enhancement of toxicity in organisms.

Noteworthily, based on the above observations, the combined effects to PS-50 and PS-500 were only observed at the upper concentration range for both plastic particles. However, the estimated environmental nanoplastic concentration for 50 nm plastic particles is speculated to be 1 pg L^–1^–15 μg/L and ≤1 μg/L for 500 nm plastic particles [20,21]. In a real environment, the environmentally relevant concentrations for the particle sizes used in the present study are most likely lower than the upper concentration. That is, from an environmental perspective, the observed combined toxicity of PS-50 and PS-500 on the functional state of the intestinal barrier may be overrated. Nevertheless, the potentially intergenerational effects of coexposure to PS-50 and PS-500 at the predicted environmental concentration cannot be neglected.

## 5. Conclusions

Taken together, coexposure to PS-50 and PS-500 was conducted to confirm our hypothesis, with the following conclusion: coexposure to micro- and nanoplastics at estimated environmentally significant concentrations could induced more severe deterioration of the functional state of the intestinal barrier than single-exposure to micro- or nanoplastics. In wild-type nematodes, cotreatment with PS-50 (15 μg/L) and PS-500 (1 μg/L) did not damage the intestinal morphology but enhanced intestinal permeability. Induction of intestinal ROS synthesis and intestinal autofluorescence production act as a cellular mechanism to powerfully explain the formation of intestine toxicity caused by coexposure to PS-50 (15 μg/L) and PS-500 (1 μg/L). Meanwhile, the downregulation of *acs-22* or *erm-1* expression and the upregulated expressions of oxidative stress-related genes serve as a molecular basis to strongly explain the formation of intestine toxicity caused by coexposure to PS-50 (15 μg/L) and PS-500 (1 μg/L). Our results suggested that combined exposure to microplastics and nanoplastics at the predicted environmental concentration notably causes intestinal toxicity by affecting the functional state of the intestinal barrier in organisms.

## Data Availability

Not applicable.

## Supplementary Materials

The following supporting information can be downloaded at https://www.mdpi.com/article/10.3390/toxics11080653/s1, Figure S1: FTIR spectrum of PS-50 and PS-500. Figure S2: Combined effects between PS-50 and PS-500 on locomotion behavior (A) and brood size (B) in nematodes. Prolonged exposure was performed from L1-larvae to adult day-1. “+”, addition; “−”, without addition. Control, without polystyrene particle exposure. Bars represent means ± SD. ** *p* < 0.01 vs. control (if not specially indicated); NS, no significant difference. Figure S3: Combined effect between PS-50 and PS-500 on intestinal permeability. The intestinal lumen (*) were labeled using asterisks. Figure S4: Combined effect between PS-50 and PS-500 on intestinal autofluorescence. Prolonged exposure was performed from L1-larvae to adult day-1. “+”, addition; “−”, without addition. Control, without polystyrene particle exposure. Bars represent means ± SD. ** *p* < 0.01 vs. control (if not specially indicated); NS, no significant difference. Figure S5: Combined effect between PS-50 and PS-500 on intestinal oxidative stress. Prolonged exposure was performed from L1-larvae to adult day-1. “+”, addition; “−”, without addition. Control, without polystyrene particle exposure. Bars represent means ± SD. ** *p* < 0.01 vs. control (if not specially indicated); NS, no significant difference. Table S1: Primers used for quantitative real-time PCR analysis.
