# Supplementary material for: Combined Effects of Micro- and Nanoplastics at the Predicted Environmental Concentration on Functional State of Intestinal Barrier in Caenorhabditis elegans
Source: Toxics. 2023 Jul 28;11(8):653. doi: 10.3390/toxics11080653 (PMC10459583; doi:10.3390/toxics11080653)
Supplement: Supplementary file 1 [file toxics-11-00653-s001.zip › toxics-2476879-supplementary.pdf]

**Supporting Information:**

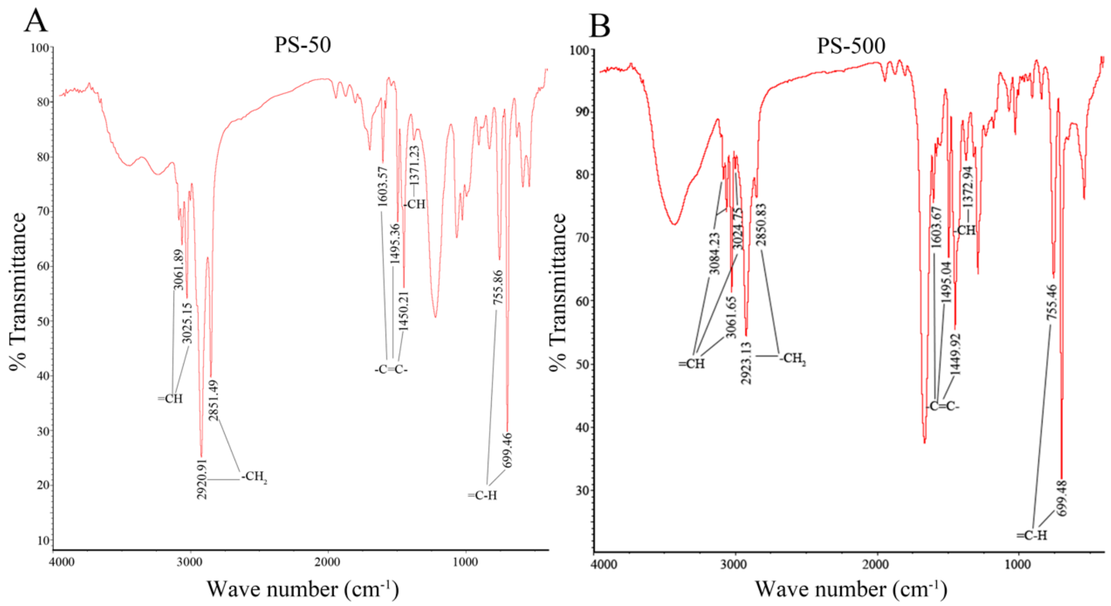


**Fig. S1.** FTIR spectrum of PS-50 and PS-500. Fourier transform infrared spectroscopy (FTIR) spectrum suggested that peaks at 2920.91 and 2851.49 cm$^{-1}$ occurred because of symmetrical bending vibration (δs), symmetric stretching vibration (σs) of methylene ($CH_2$) for PS-50 (A), 2923.13 and 2850.83 cm$^{-1}$ for PS-500 (B); a peak at 3061.89 and 3025.15 cm$^{-1}$ attributed to the stretching vibration (σ) of unsaturated hydrocarbon group on benzene ring (=CH) for PS-50 (A), 3084.23 and 3061.65 cm$^{-1}$ for PS-500 (B); peaks at 1603.57, 1495.36, and 1450.21 cm$^{-1}$ were generated by benzene ring skeleton vibration (δ) (-C=C-) for PS-50 (A), 1603.67, 1495.04, and 1449.92 cm$^{-1}$ for PS-500 (B); peaks at 755.86 and 699.46 cm$^{-1}$ were due to out-of-plane bending vibration (δ) of unsaturated hydrocarbon groups on benzene ring (=C-H) for PS-50 (A), 755.46 and 699.48 cm$^{-1}$ for PS-500 (B).

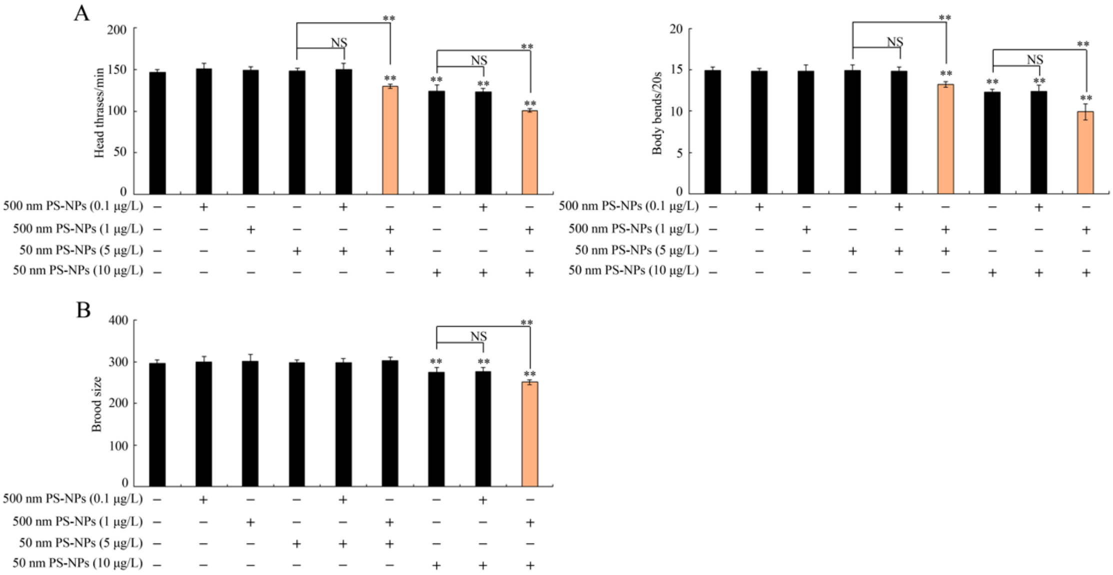


**Fig. S2.** Combined effects between PS-50 and PS-500 on locomotion behavior (A) and brood size (B) in nematodes. Prolonged exposure was performed from L1-larvae to adult day-1. "+", addition; "-", without addition. Control, without polystyrene particle exposure. Bars represent means ± SD. **P < 0.01 vs control (if not specially indicated); NS, no significant difference.

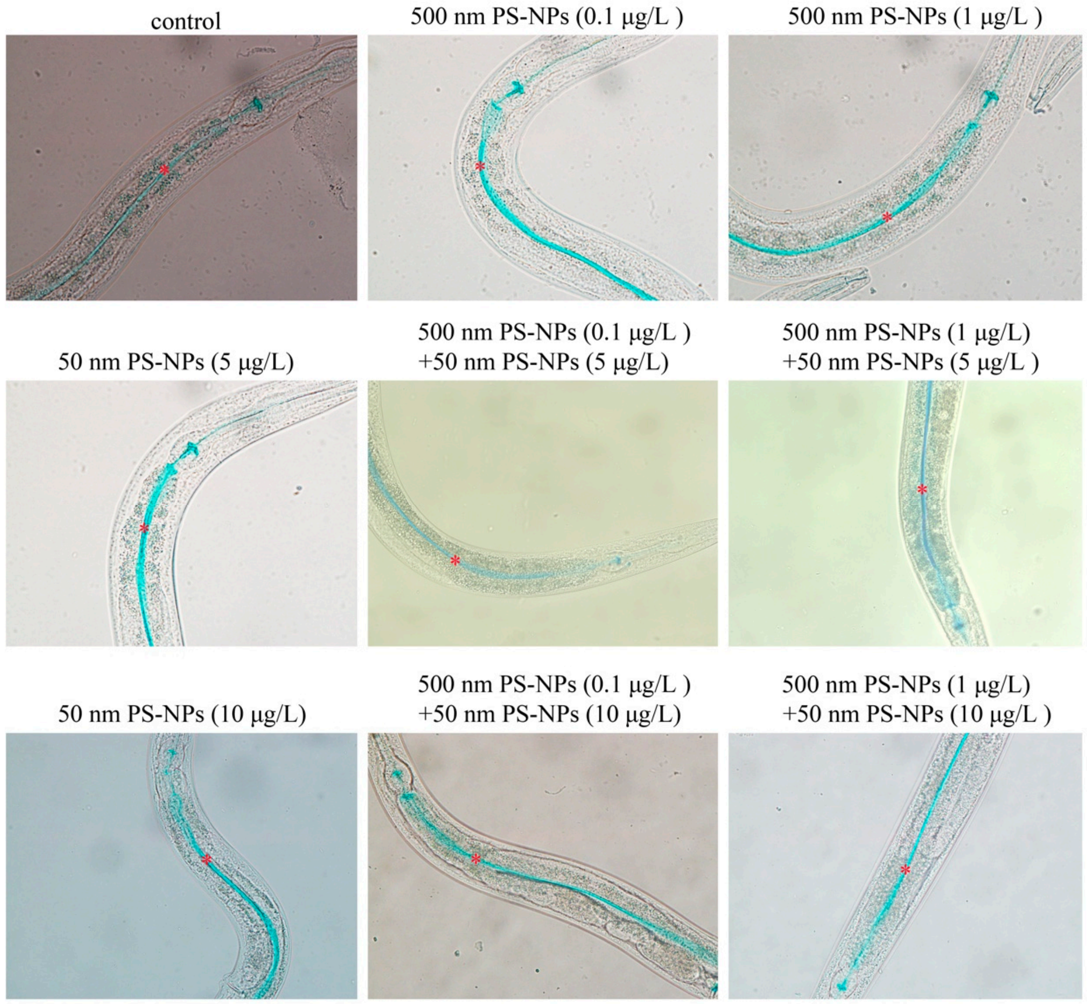


**Fig. S3.** Combined effect between PS-50 and PS-500 on intestinal permeability. The intestinal lumen (*) were labeled by asterisks.

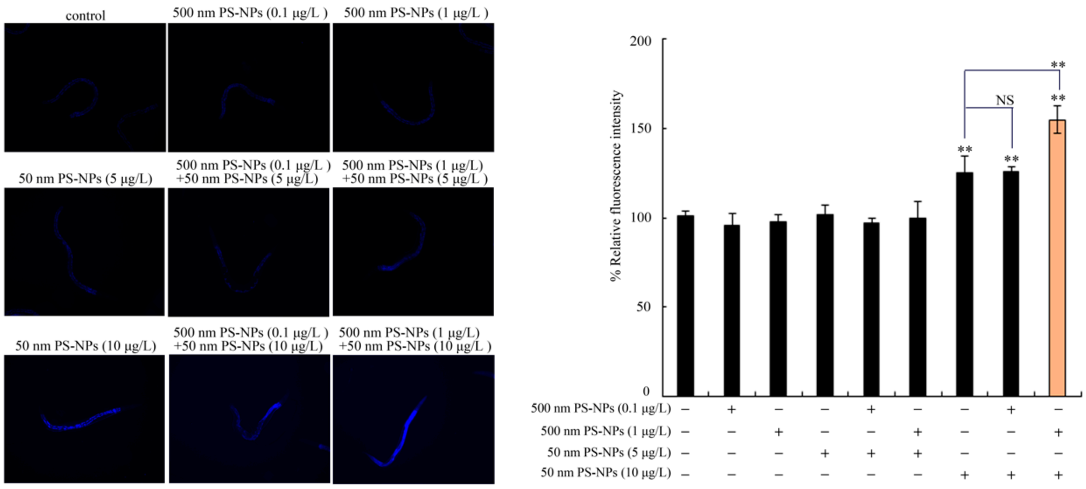


**Fig. S4.** Combined effect between PS-50 and PS-500 on intestinal autofluorescence. Prolonged exposure was performed from L1-larvae to adult day-1. "+", addition; "-", without addition. Control, without polystyrene particle exposure. Bars represent means ± SD. $^{**}P < 0.01$ vs control (if not specially indicated); NS, no significant difference.

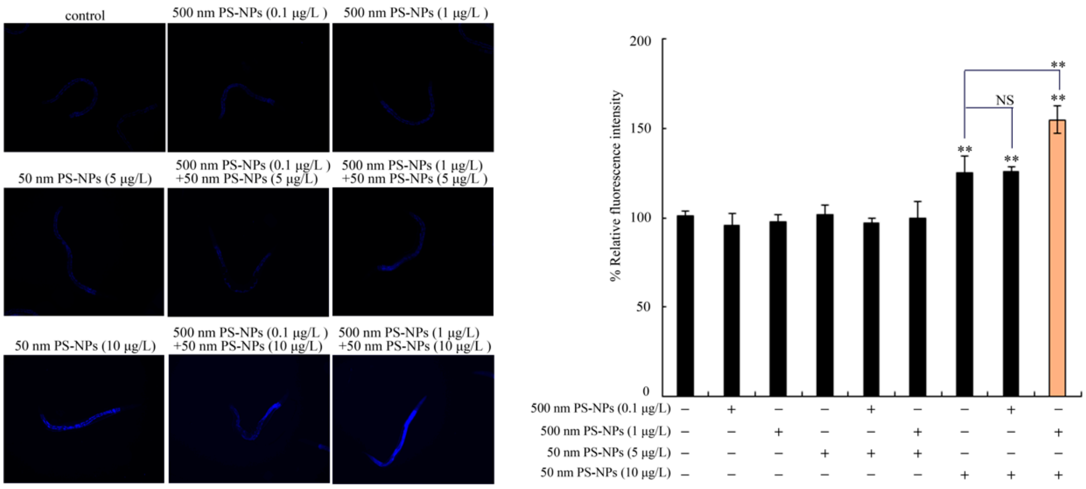


**Fig. S5.** Combined effect between PS-50 and PS-500 on intestinal oxidative stress. Prolonged exposure was performed from L1-larvae to adult day-1. "+", addition; "-", without addition. Control, without polystyrene particle exposure. Bars represent means ± SD. **P < 0.01 vs control (if not specially indicated); NS, no significant difference.

**Table S1.** Primers used for quantitative real-time PCR analysis

| Gene | Forward Primer (5′-3′) | Reverse Primer (5′-3′) |
|---|---|---|
| *tba-1* | TCAACACTGCCATCGCCGCC | TCCAAGCGAGACCAGGCTTCAG |
| *sod-1* | ACGCTCGTCACGCTTTAC | TCTTCTGCCTTGTCTCCG |
| *sod-2* | GGCATCAACTGTCGCTGT | ACAAGTCCAGTTGTTGCC |
| *sod-3* | TGACATCACTATTGCGGT | GGGACCATTCCTTCCAAA |
| *sod-4* | CACCAGATGACTCGAACA | AATGAGGCAAGAGAGTCG |
| *sod-5* | AAAGTAGAGTCGAAACGTGCTG | TGAAGTCCTGGTGACAATCCCT |
| *clk-1* | CACATACTGCTGCTTCTCGT | TGAACCAACAGATGAACCTT |
| *gas-1* | CTTGGTCTTTGGCTGTTGA | CTTGGTCTTTGGCTGTTGA |
| *isp-1* | GCAGAAAGATGAATGGTCC | CAGAAGCGTCGTAGTGAGA |
| *mev-1* | GGAATTCGCTTCTTAGGAT | GCAGTCTTGTTGCTCTTGT |
| *ctl-1* | TGTCGTTCATGCCAAGGGAG | GATCCCGATTCTCCAGCGAC |
| *ctl-2* | TCCCAGATGGGTACCGTCAT | GGTCCGAAGAGGCAAGTTGA |
| *ctl-3* | ATGCCAATGCTTCCCCACAT | ACGGCGGTCTTCGAGTAGAT |
| *act-5* | CCTGCTTGGAGATCCACATT | CACCCAGTTCTCCTTACCGA |
| *erm-1* | CAGAACGTCGTAGTCAGTGACAG | TTTCAGGACTTTGTCTTCTACGC |
| *pkc-3* | CATTTCCAACCACAATTCCC | TGTTCCAAAGCTTCCCAATC |
| *hmp-2* | TTCCGGTGGTTCAAAGTTTC | GATGGCAGCTGAATCTCCTC |
| *acs-22* | TCATGCCAATTTATCCCCAT | AAATGAGCCGAGAGGGAAAT |
